# Efficient gradient calibration based on diffusion MRI

**DOI:** 10.1002/mrm.26105

**Published:** 2016-01-08

**Authors:** Irvin Teh, Mahon L. Maguire, Jürgen E. Schneider

**Affiliations:** ^1^ Division of Cardiovascular MedicineRadcliffe Department of Medicine, University of OxfordOxfordUnited Kingdom; ^2^ British Heart Foundation (BHF) Centre of Regenerative MedicineOxfordUnited Kingdom

**Keywords:** gradient calibration, gradient nonlinearity, diffusion MRI, quantitative MRI, cyclooctane

## Abstract

**Purpose:**

To propose a method for calibrating gradient systems and correcting gradient nonlinearities based on diffusion MRI measurements.

**Methods:**

The gradient scaling in x, y, and z were first offset by up to 5% from precalibrated values to simulate a poorly calibrated system. Diffusion MRI data were acquired in a phantom filled with cyclooctane, and corrections for gradient scaling errors and nonlinearity were determined. The calibration was assessed with diffusion tensor imaging and independently validated with high resolution anatomical MRI of a second structured phantom.

**Results:**

The errors in apparent diffusion coefficients along orthogonal axes ranged from −9.2% ± 0.4% to + 8.8% ± 0.7% before calibration and −0.5% ± 0.4% to + 0.8% ± 0.3% after calibration. Concurrently, fractional anisotropy decreased from 0.14 ± 0.03 to 0.03 ± 0.01. Errors in geometric measurements in x, y and z ranged from −5.5% to + 4.5% precalibration and were likewise reduced to −0.97% to + 0.23% postcalibration. Image distortions from gradient nonlinearity were markedly reduced.

**Conclusion:**

Periodic gradient calibration is an integral part of quality assurance in MRI. The proposed approach is both accurate and efficient, can be setup with readily available materials, and improves accuracy in both anatomical and diffusion MRI to within ±1%. Magn Reson Med 77:170–179, 2017. © 2016 The Authors Magnetic Resonance in Medicine published by Wiley Periodicals, Inc. on behalf of International Society for Magnetic Resonance in Medicine.

## INTRODUCTION

Accurate gradient calibration is a prerequisite for quantitative measurements in MRI and spectroscopy in general. Miscalibrated gradients can, for instance, lead to systematic over‐ or underestimation in geometric measurements. Calibration is usually performed by vendors at installation and during routine servicing, based on anatomical scans of a phantom of known dimensions. Phantoms with more complex geometries, typically grid structures over an extended field of view (FOV), have also been proposed for gradient calibration, with the added benefit of addressing gradient nonlinearities 1, 2, 3. The accuracy of these approaches is governed by the spatial resolution of the scan, and the geometric accuracy of the phantom dimensions.

Diffusion MRI is particularly sensitive to poorly calibrated gradients as the measured apparent diffusion coefficient (ADC) depends on the square of the gradient amplitude. A typical error of ±2% in gradient strength, for example, would lead to a ±4% error in the measured ADC 4. Differential errors between the x‐, y‐, and z‐gradients would additionally lead to errors in fractional anisotropy (FA) and eigenvector estimates, as they depend on the sample diffusion orientation with respect to the diffusion‐weighting direction. The corollary is that the measured diffusion can provide a sensitive means for gradient calibration. Fluid‐filled phantoms are well suited for this purpose, and corrections can be determined on the basis that diffusion in such phantoms is isotropic and Gaussian. Examples include the use of phantoms filled with water 5, 6, polyvinylpyrolidone 7, ethylene glycol 8, *n*‐undecane 9, and dodecane 10. Use of such phantoms benefits from ease of preparation and access to reliable source materials. However, as diffusion is dependent on temperature, these methods require either accurate control or monitoring of temperature, such as with an ice‐water phantom 11, temperature measurement before and/or after scanning 6, 9, periodic temperature sampling with MR spectroscopy 8, and real‐time temperature monitoring with a thermistor 12. A criticism of water‐based phantoms is their relative low viscosity renders them susceptible to vibration, convection and flow 9, 13, and their high diffusivity limits the use of higher b‐values. In contrast, more viscous media such as cyclooctane and ethylene glycol have been shown to exhibit monoexponential behavior up to a b‐value of 10,000 and 12,000 s/mm^2^, respectively 8, 12. Cyclooctane has the added benefit of having single proton resonance, thereby avoiding chemical shift artifacts and signal cancellation from J‐coupling.

Depending on the calibration method employed, different means of assessing gradient calibration performance have been proposed. Studies that derive image deformation maps from high‐resolution distortion‐free reference x‐ray CT data typically measure improvement in the conformance between the MRI and CT images after calibration 1, 3. In studies of diffusion‐based calibration methods, improvements were shown in the reduced directional bias in gradient strengths reflected in lower FA 5, 9, 10 and more robust fiber tracking 6, 10.

The aim of this study was to propose a simple and efficient method for calibrating the gradient scaling in x, y, and z, based on a phantom with well‐characterized diffusivity and constructed from readily available materials. The same principle can be extended to correct for gradient nonlinearity in a model‐free manner. The improvements in gradient calibration will lead to improved accuracy and precision in quantitative MRI. Such improvements were demonstrated with diffusion MRI in the same phantom, and independently validated with high‐resolution anatomical MRI in a second custom‐built grid phantom.

## METHODS

### Phantom Design

**Figure 1:**
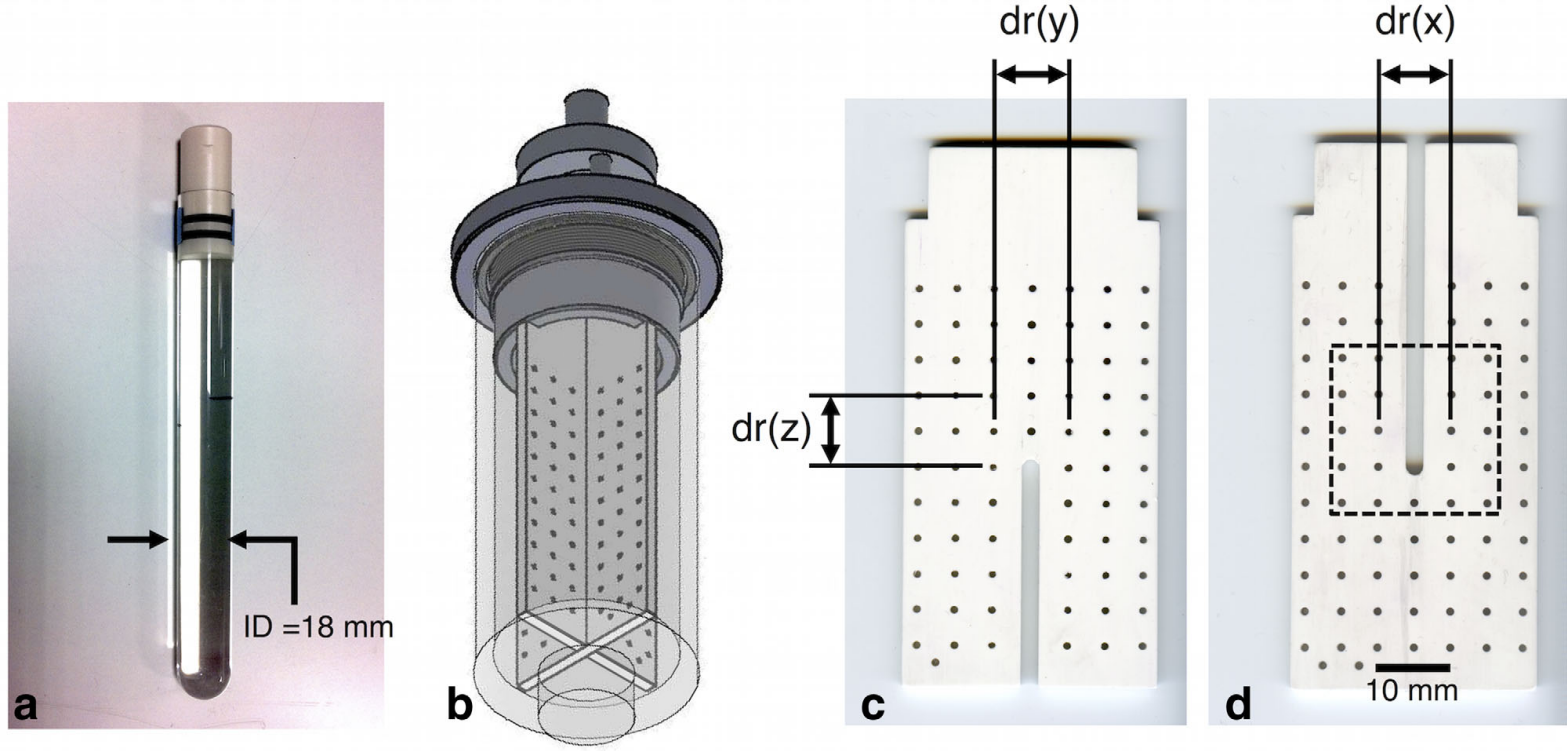
(**a**) Photograph of the diffusion calibration phantom comprising a sealed tube of cyclooctane. (**b**) Schematic of the grid phantom for geometric validation shows two orthogonal slotted plates in a cylindrical housing containing 2 mM aqueous gadolinium solution. (**c, d**) Prior to assembly, the two plates were scanned with a desktop flatbed optical scanner to obtain reference geometric information. The dotted square identifies the region in which the measured and reference centroid maps were overlaid as in Figure 3. Reference distances *dr*(*x*), *dr*(*y*), and *dr*(*z*) are indicated and reported in Table 1.

A diffusion calibration phantom was constructed by filling a 20‐mm outer diameter glass tube with 99% cyclooctane (Sigma‐Aldrich, St. Louis, Missouri, USA) while avoiding air bubbles. The tube was sealed with a polyphenylsulphide plug and two Viton O‐rings (Fig. 1a). A thermistor embedded in epoxy resin, used for monitoring of temperature in small animals, was connected to a Harvard Apparatus homeostatic temperature control unit (Harvard Apparatus, Kent, United Kingdom). The thermistor was calibrated and secured to the surface of the tube, and the temperature was recorded at 1 Hz on a Powerlab/30 using Chart v5.0 (AD Instruments, Bella Vista, New South Wales, Australia). A second grid phantom for geometric validation comprised two orthogonal 2‐mm‐thick slotted plates of Tecapet (Ensinger, Nufringen, Germany) that fitted in a cylindrical housing made from polyvinylchloride (Trovex Diamond, Hertfordshire, UK) (Fig. 1b). A grid pattern of 1‐mm‐diameter holes was drilled at nominally 5‐mm intervals (Fig. 1c,d) and the phantom was filled with 2.0 mM aqueous gadolinium solution (Prohance; Bracco Diagnostics Inc, Cranbury, New Jersey, USA).

### Diffusion MRI Acquisition

The calibration scans were performed using a 9.4 T preclinical scanner and a shielded gradient system (Agilent Technologies, Santa Clara, California, USA). The inner diameter of the gradient set was 60 mm, and the maximum gradient strength was 1 T/m with a rise time of 130 μs. A quadrature‐driven transmit/receive birdcage radiofrequency coil of 20 mm inner diameter and coil sensitivity of 25 mm in z was used (Rapid Biomedical, Rimpar, Germany). Prior to the study, a rough calibration was performed. To simulate a poorly calibrated system, the gradient scaling factors in x and z were first offset by −5% and +5% from the precalibrated values. Data were acquired with diffusion‐weighting in x, y, and z separately using two‐dimensional (2D) spin echo (SE) echo planar imaging with pulsed gradient SE diffusion‐weighting 14. The sequencing parameters were as follows: repetition time (TR)/echo time (TE) = 2000/20 ms; echo train length = 16; resolution = 375 × 375 μm; FOV = 24 × 24 mm; slice thickness = 1 mm; number of signal averages (NSA) = 4; number of slices = 11; δ = 2.5 ms; Δ = 15 ms; b = [100, 400, 900, 1600, 2500] s/mm^2^; and acquisition time = 5 min, 36 s. Forward and reverse readout polarity data were acquired to correct for errors between odd and even lines of k‐space 15. To minimize the b‐value contribution from imaging gradients, refocusing crushers were omitted. The b‐values specified included contributions from imaging and cross‐terms, and the diffusion gradient strength was adjusted accordingly 16. Diffusion tensor imaging (DTI) was performed with a 2D SE echo planar imaging sequence, and FA was measured in five central axial slices 17. Scan parameters were similar to the calibration scan with the following differences: number of non–diffusion‐weighted scans = 3, number of diffusion directions = 21 18, b‐value = 2000 s/mm^2^. Data with forward and reverse diffusion gradient polarity were acquired to correct for the effect of cross‐terms 19. Correction factors were calculated and were used to adjust the gradient scaling. The calibration and DTI scans were repeated in the same experiment postcalibration.

In a separate experiment, 2D SE data were acquired to correct for gradient nonlinearity. The data were acquired postcalibration, and with diffusion‐weighting in x, y, and z separately. A quadrature‐driven transmit/receive birdcage coil of 28 mm inner diameter (Rapid Biomedical, Rimpar, Germany) and coil sensitivity of 45 mm in z was used. The sequence parameters were as follows: TR/TE = 5000/65 ms; resolution = 100 × 100 μm; FOV = 57.6 × 19.2 mm; slice thickness = 1 mm; NSA = 1; number of slices = 1; δ = 2.5 ms; Δ = 15 ms; b = [100, 400, 900, 1600, 2500] s/mm^2^, and acquisition time = 1 h, 20 min per diffusion‐weighted (DW) direction. Data were acquired in sagittal and coronal planes.

### Correction for Gradient Linear Scaling

The measured ADCs, *D*_*m*_(*i*), were first calculated by performing a linear fit of the ln signal intensity in the calibration data along the individual DW directions, *i*, and taking the mean over a central 7 × 7 × 7 voxel region. This signal behavior is described by

(1) $$S=S_{0}exp^{-b\left(i\right)*D_{m\;}\left(i\right)},$$

where *S* is the measured signal intensity at the applied b‐value, *b*, and *S*_*0*_ is the non–diffusion‐weighted signal intensity.

The raw temperature data were smoothed with a sliding window method (mean temperature within a 60‐s interval) to reduce noise. Temperature readings, *T*(*a,b,i*) corresponding to each average, b‐value, and DW direction were obtained. Matching reference ADCs, *D*_*r*_(*a,b,i*), were calculated at *T*(*a,b,i*) by fitting a second‐order polynomial to a range of reference diffusivity data 13 and averaged across NSA and b‐values to obtain *D*_*r*_(*i*). Correction factors, *α*(*x*), *α*(*y*) and *α*(*z*) were calculated using Equation (2) and derived here 6; corrected gradient scaling factors, *Ψ'*(*x*), *Ψ'*(*y*), and *Ψ'*(*z*) were calculated with Equation (3) and applied, where *Ψ*(*i*) is the uncorrected gradient scaling factor:

(2) $$\alpha \left(i\right)=\sqrt{\left(\frac{D_{r}\left(i\right)}{D_{m}\left(i\right)}\right)}$$

(3) Ψ′(i)= α(i)* Ψ(i).

To assess the effect of NSA and b‐values sampled for the gradient calibration, correction factors were also calculated after subsampling *D*_*m*_(*i*) and *D*_*r*_(*i*) by number of averages (1, 2, 3, and 4) and number of b‐values used (2, 3, 4, and 5). The b‐value combinations used were [100, 2500], [100, 900, 2500], [100, 400, 900, 2500], and [100, 400, 900, 1600, 2500] s/mm^2^ respectively. The effective scan times ranged from 48 s to 5 min, 36 s.

The effect of cross‐terms was removed from the DTI data by taking the geometric mean of data acquired with opposing diffusion gradient polarities 19, and the results were fit with a single tensor and linear least squares. The tensors were diagonalized and the FA 20 was calculated in each voxel based on Equation (4), where *λ*_*1*_, *λ*_*2*_, and *λ*_*3*_ are the eigenvalues of the tensor:

(4) $$\text{FA}=\frac{1}{\sqrt{2}}\frac{\sqrt{{\left(\lambda_{1}-\lambda_{2}\right)}^{2}+{\left(\lambda_{2}-\lambda_{3}\right)}^{2}+{\left(\lambda_{3}-\lambda_{1}\right)}^{2}}}{\sqrt{\lambda_{1}^{2}+\lambda_{2}^{2}+\lambda_{3}^{2}}}.$$

### Correction for Gradient Nonlinearities

To correct for gradient nonlinearities, *D*_*m*_(*x,y,z,i*) were first calculated by performing a linear fit of the ln signal intensity in the SE DW data along the individual DW directions, *i*, on a voxel‐wise basis. *α* correction maps were generated using Equation (2). Instead of adjusting the gradient scaling, deformations were calculated from the isocenter in 2D according to Equation (5) and applied to the image data based on the correction maps. The integral of the signal intensity per unit voxel area was preserved postcorrection for gradient nonlinearity according to Equation (6). The corrected image data were then resampled to the coordinate space of the original data and were reprocessed to obtain ADC and *α* correction maps.

(5) $$\left[\begin{array}{c} p_{m}\\ p_{n} \end{array}\right]=\left[\begin{array}{ccc} \alpha_{r,m} & 0 & p_{m-1}\\ 0 & \alpha_{p,n} & p_{n-1} \end{array}\right]\;\left[\begin{array}{c} dr\\ dp\\ 1 \end{array}\right]$$

(6) $$S_{m,n}^{*}=\frac{S_{m,n}}{\alpha_{r,m}*\alpha_{p,n}}$$

Here, ***p*** is a vector of coordinates reflecting the corrected voxel positions in the readout (*r*) and phase encoding (*p*) directions; *m* and *n* are voxels measured from the isocenter outward along *r* and *p* and range from 1 to 288 and 1 to 96, respectively, or half the matrix size along the respective directions; ***α*** represents the correction factors calculated in *r* and *p* directions at the corresponding voxels; *dr* and *dp* are nominal voxel dimensions in *r* and *p* directions; and ***S*** and S* are vectors of signal intensity in the uncorrected and corrected image data. For illustration purposes, the corrections for gradient nonlinearities were first applied in the phase encoding direction, and subsequently in the readout direction.

To correct for the effects of gradient nonlinearities on the measured ADCs, the b‐matrices were recalculated on a voxel‐wise basis after dividing the magnitude of the gradient waveforms (including diffusion and imaging gradients) by *α* from the unwarped correction maps. The b‐matrices were calculated numerically and included contributions from the diffusion and imaging gradients and cross‐terms 21. The measured ADCs, *D*_*m*_(*x,y,z,i*), were calculated after correction for gradient nonlinearities as before.

### Validation

Prior to assembly, reference geometric data were obtained by scanning the two plates of the grid phantom with an Epson Perfection V370 desktop flatbed optical scanner (Epson, Nagano, Japan) at 5.3 × 5.3 μm resolution. The phantom was then scanned in a transmit/receive birdcage coil of 42 mm inner diameter and coil sensitivity of 55 mm in z (Rapid Biomedical, Rimpar, Germany) with three‐dimensional (3D) SE MRI before and after gradient calibration to obtain geometric measurements. The scanning parameters were as follows: TR/TE = 80/9.2 ms; resolution = 100 × 100 × 100 μm; FOV = 65.0 × 38.4 × 38.4 mm; and acquisition time = 3 h, 17 min. Central sagittal and coronal planes in the MRI data corresponding to the two plates were selected and interpolated to 10 × 10 μm resolution by zero‐filling in k‐space. The holes forming the grid were automatically segmented, and their centroids were detected in both MRI and optical scan data. Distances along x, y, and z between the two holes adjacent to the center‐most hole in the MRI data, *dm*(*x*), *dm*(*y*), and *dm*(*z*), were measured and compared with the reference data from the high resolution optical scan, *dr*(*x*), *dr*(*y*) and *dr*(*z*) (Fig. 1c,d). In addition, the correction for gradient nonlinearity determined from the 2D SE data was applied to the matching cropped sagittal and coronal views in the grid phantom data. For clarity, the corrections for gradient nonlinearities were again first applied in the phase encoding direction, and subsequently in the readout direction. All data analysis was performed in MATLAB 2013a (MathWorks, Natick, Massachusetts USA).

## RESULTS

### Correction for Gradient Linear Scaling

**Figure 2:**
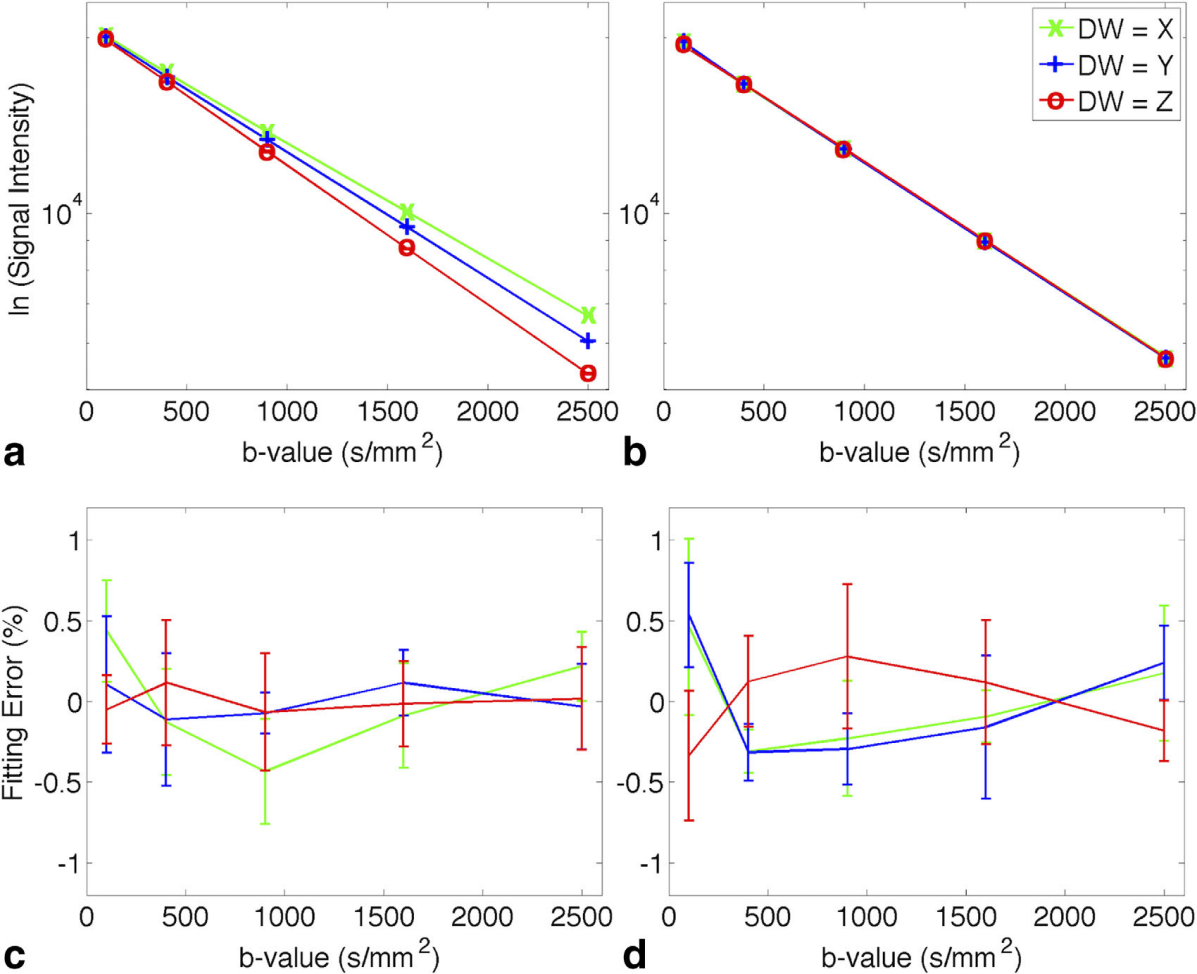
(**a, b**) Semi‐log diffusion signal attenuation curve in the (a) precalibration and (b) postcalibration data. A linear function was used to fit the ln(signal intensity) and R^2^ > 0.999 for all fits. The data shown (mean ± standard deviation) were reported in a central 7 × 7 × 7 voxel region (corresponding to a volume of 2.6 × 2.6 × 7.0 mm) across scan repetitions (NSA = 4); all standard deviations < 106 and the error bars are smaller than the plot symbols. The higher and lower gradient of the DW = Z and DW = X lines in the precalibration data reflect the positive and negative offset in the diffusion gradient strength. This was corrected postcalibration. (**c, d**) Percentage fitting errors calculated as (S_measured −_ S_fit_)/S_fit_ · 100% are shown for the (c) precalibration and (d) postcalibration data.

The measured ADC, as determined from the gradient of the ln signal versus b‐value curve, shows marked differences between DW directions prior to calibration; these differences were corrected following calibration (Fig. 2). The errors in precalibration *D*_*m*_(*x,y,z*) were −9.2% ± 0.4%, −1.1% ± 0.5%, and +8.8% ± 0.7% with respect to the reference values. After correction for linear scaling, the errors in *D*_*m*_(*x,y,z*) were −0.5% ± 0.4%, + 0.8% ± 0.3%, and −0.1% ± 0.7% with respect to the reference values (R^2^ > 0.999 for all fits). The percentage fitting errors between the measured signal and the fitted ADC remained <0.53% across all b‐values.

**Figure 3:**
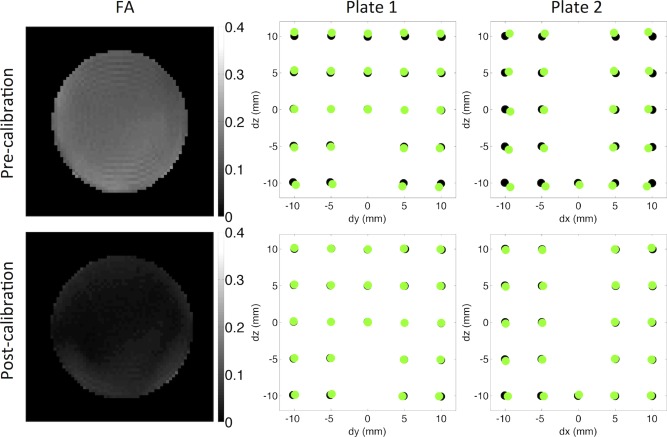
Left column: The precalibration and postcalibration FA maps show that the FA is overestimated precalibration and approaches zero postcalibration. Middle and right columns: The centroids of the holes calculated from the 3D SE MRI data (green) and the reference optical scan data (black) are overlaid in a central 24 × 24 mm region as shown in Figure 1. These exhibited improved correspondence postcalibration.

FA was elevated prior to calibration as seen in a central axial slice of the calibration phantom; this elevation in FA was reduced following calibration (Fig. 3). Figure 3 also shows the centroids of the holes identified from the anatomical MRI overlaid on those identified with the reference optical scan in a central 24 × 24 mm region. Prior to calibration, it can be seen that the MRI data are compressed in x and stretched in z relative to the optical scan data. These scalings are the result of reduced and elevated gradient scaling in x and z, respectively, as reflected in the low *D*_*m*_(*x*) and high *D*_*m*_(*z*) with respect to the calibrated diffusion data. The gradient scaling errors resulted in an artifactually larger FOV in x and smaller FOV in z; consequently, there was an apparent compression in x and stretching in z. The correspondence of MRI and optical scan data is significantly improved in both plates of the grid phantom postcalibration.

The diffusion measurements, correction factors, and geometric validation are summarized in Table 1. The mean temperature was 20.6°C ± 0.2°C and 21.3°C ± 0.04°C in the precalibration and postcalibration scans, respectively. In addition to the improved estimation of *D*_*m*_(*i*) with respect to *D*_*r*_(*i*) postcalibration, we observed that the FA decreased from 0.14 ± 0.03 to 0.03 ± 0.01 following calibration, better representing the isotropic fluid. The range of calculated correction factors was reduced from 0.959 to 1.050 before calibration to 0.996 to 1.003 after calibration. Similarly, the differences in geometric measurements *dm*(*i*) with respect to the optical scan reference data *dr*(*i*) ranged from −5.5% to + 4.5% before calibration and −0.97% to + 0.23% after calibration.

**Table 1:** Measured and Reference Apparent Diffusion Coefficients (*D*_*m*_ and *D*_*r*_), Correction Factors (*α*) and Geometric Measurements (*dm* and *dr*) in x, y, and z Directions Before and After Calibration

	Parameter	Units	Grid Phantom	Diffusion Phantom Precalibration	Diffusion Phantom Postcalibration
Diffusion measurements	*D*_*m*_(*x*)	×10^−4^ mm^2^/s		4.58 ± 0.02	5.11 ± 0.02
	*D*_*m*_(*y*)			4.96 ± 0.02	5.17 ± 0.02
	*D*_*m*_(*z*)			5.48 ± 0.03	5.12 ± 0.04
	*D*_*m*_(mean)			5.01 ± 0.39	5.13 ± 0.04
	*D*_*r*_(*x*)			5.04 ± 0.01	5.135 ± 0.002
	*D*_*r*_(*y*)			5.019 ± 0.004	5.129 ± 0.004
	*D*_*r*_(*z*)			5.04 ± 0.03	5.13 ± 0.01
	*D*_*r*_(mean)			5.03 ± 0.02	5.13 ± 0.01
	FA			0.14 ± 0.03	0.03 ± 0.01
Correction factors	*α*(*x*)			1.05	1.003
	*α*(*y*)			1.006	0.996
	*α*(*z*)			0.959	1.001
Geometric Validation	*dm*(*x*)	mm		9.46 (−5.45%)	9.91(−0.97%)
	*dm*(*y*)			9.93 (−1.14%)	9.98 (−0.64%)
	*dm*(*z*)			10.39 (4.46%)	9.97 (−0.23%)
	*dr*(*x*)		10.01		
	*dr*(*y*)		10.04		
	*dr*(*z*)		9.94		

Percentage differences between mean measured and reference values are given in parentheses. All diffusion measurements including FA and geometric measurements are closer to the reference values postcalibration.

**Figure 4:**
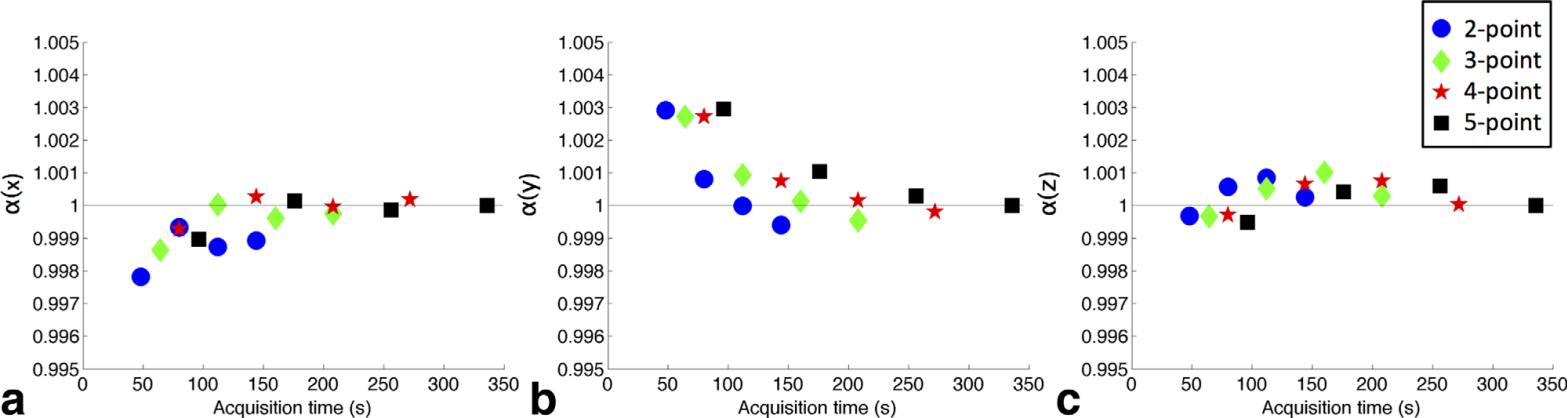
Correction factors (**a**) *α*(*x*), (**b**) *α*(*y*), and (**c**) *α*(*z*) were calculated based on subsampling the number of signal averages (NSA) and the number of points on the diffusion signal attenuation curve. These were normalized to the NSA = 4, 5‐point calibration data. Each scheme (2 ≤ n ≤ 5) is plotted over a range of averages (1 ≤ NSA ≤ 4) data from the left to the right in each graph. The estimation of *α* at an effective scan time of 48 s remained within 0.3% of *α* calculated based on the fully sampled data with a scan time of 5 min, 36 s.

The correction factors, *α*(*i*), were originally calculated based on data acquired with NSA = 4 and 5 b‐values. Figure 4 illustrates the effect of subsampling NSA and the number of b‐values used to calculate *α*(*i*) to potentially reduce data acquisition requirements. The normalized results show that calculating *α*(*i*) based on NSA = 2 and 2 b‐values yielded differences of < 0.1% from the nominal *α*(*i*), and calculating *α*(*i*) based on NSA = 1 and 2 b‐values yielded differences of < 0.3%. These data could be acquired in 48 s and 80 s, respectively, where the NSA = 4 and 5 b‐values data required 5 min, 36 s.

### Correction for Gradient Nonlinearities

**Figure 5:**
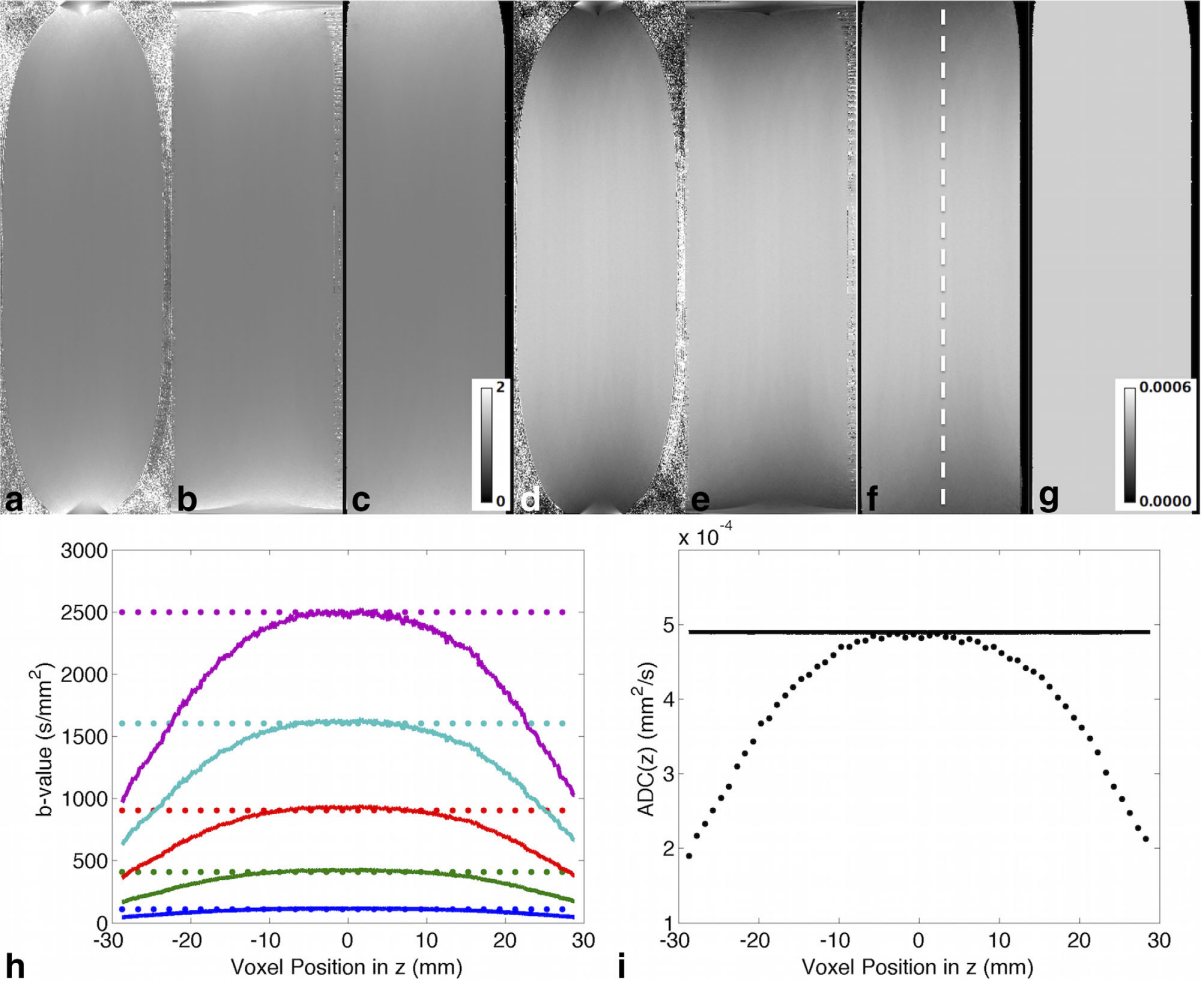
(**a–c**) *α* correction maps in the diffusion calibration phantom, with diffusion along the readout direction (z) and in coronal view (a) without correction for gradient nonlinearity, (b) with correction in the phase encoding direction (x) only, and (c) with correction in the phase encoding and readout directions. (**d–f**) Corresponding apparent diffusion coefficient (ADC) maps (mm^2^/s). The cylindrical geometry of the tube is recovered postcorrection. The estimated *α* increases and ADC decreases toward either end of the coil, where the gradient profile becomes increasingly nonlinear. (**g**) The corrected ADC map (mm^2^/s) is more homogeneous after correction. (**h**) Nominal (dotted) and corrected (solid) b‐values across a profile in z as indicated by the dashed line in panel F. Nominal b = 100, 400, 900, 1600, and 2500 s/mm^2^ are shown in blue, green, red, cyan, and magenta, respectively. (**I**) Nominal (dotted) and corrected (solid) ADC in z across the same profile along z. The nominal ADC was subsampled by a factor of 10 for display purposes, to distinguish it from the corrected data.

The effects of the correction for gradient nonlinearity on the ADC and *α* are shown (Fig. 5a–f). While *α* is relatively uniform near the isocenter, it rapidly increases toward the ends of the phantom along z. This increase is accompanied by a concomitant narrowing of the appearance of the tube diameter. The geometric accuracy of the image data improves with stepwise corrections in the phase encoding and readout directions, as reflected in the more cylindrical appearance of the phantom. Regions of higher *α* corresponded to regions with lower ADC and vice versa. The results from the data acquired in the sagittal and coronal views were similar; for brevity, only data acquired in the coronal view are presented.

As the gradient strength decreases further away from the magnet isocenter, so does the effective b‐value. This is described in a plot of the nominal and effective b‐value profiles along z (Fig. 5h). Recalculation of the ADC based on the effective b‐values shows the effect of gradient nonlinearity correction across a profile in z (Fig. 5i) and in a coronal 2D image (Fig. 5g).

**Figure 6:**
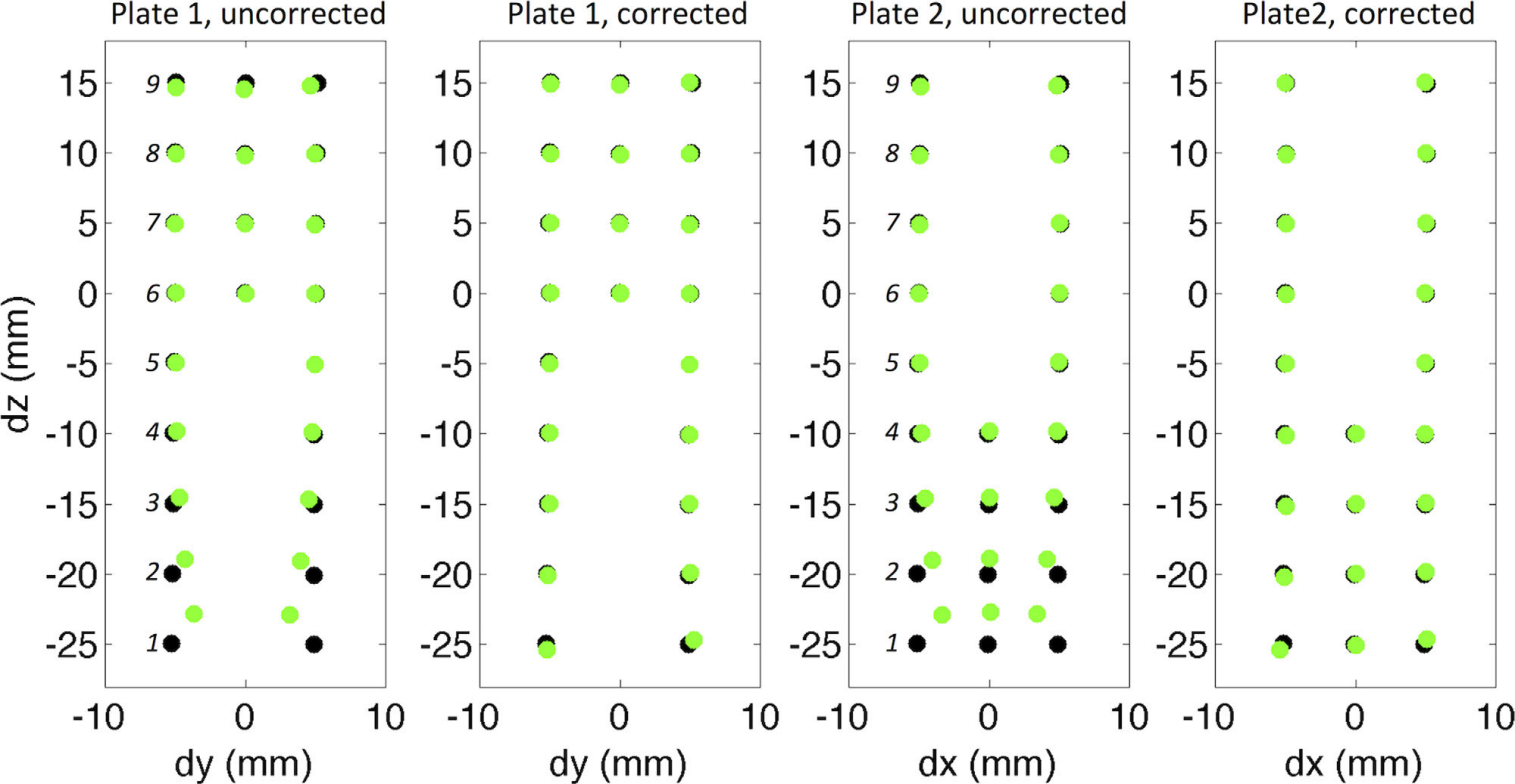
The correspondence of the centroids of the holes calculated from the 2D SE MRI data (green) and the reference optical scan data (black) are improved in both plates after correction for gradient nonlinearity. Errors in the physical coordinates of centroids 1–9 (in italics) are summarized in Table 2.

Overlaying the centroids of the holes in the 2D SE MRI data with that of the reference optical scan data shows much better correspondence after correction for gradient nonlinearity (Fig. 6). Nine centroids were identified in each of the two plates, and the errors in their physical coordinates in x, y, and z with respect to the reference optical scan data are presented in Table 2. At point 1, the furthest identified point from the magnet isocenter, the absolute error in x, y and z coordinates were 35%, 31% and 8.8% in the uncorrected data and 4.9%, 1.5% and 1.6% in the corrected data. At point 2, these values were 21%, 18%, and 5.5% and 1.7%, 1.5%, and 0.5%, respectively. Supporting research data are available upon request.

**Table 2:** Errors in the Physical Coordinates of Centroids 1–9, as Identified in Figure 6, Between the MRI Measurements *dm*(*x*), *dm*(*y*), and *dm*(*z*) and Reference Optical Scan Measurements *dr*(*x*), *dr*(*y*), and *dr*(*z*)

		1	2	3	4	5	6	7	8	9
Uncorrected	*dm*(*x*) − *dr*(*x*)	1.79	1.07	0.55	0.27	0.11	0.01	0.03	0.02	0.06
	*dm*(*y*) − *dr*(*y*)	1.62	0.91	0.46	0.25	0.13	0.05	0.09	0.05	0.01
	*dm*(*z*) − *dr*(*z*)	2.20	1.10	0.50	0.11	−0.05	−0.02	−0.04	−0.14	−0.40
Corrected	*dm*(*x*) − *dr*(*x*)	−0.25	0.09	0.14	0.13	0.09	0.05	0.07	0.01	−0.05
	*dm*(*y*) − *dr*(*y*)	0.08	0.08	0.11	0.12	0.11	0.08	0.12	0.02	−0.03
	*dm*(*z*) − *dr*(*z*)	−0.40	−0.10	0.00	−0.05	−0.12	−0.01	−0.02	−0.10	−0.20

*dm*(*x*) and *dr*(*x*) are cited based on plate 2, whereas *dm*(*y*), *dm*(*z*), *dr*(*y*), and *dr*(*z*) are based on plate 1. Distances are measured in millimeters.

## DISCUSSION

The common approach of calibrating gradients based on anatomical MRI of a phantom with known dimensions assumes gradient linearity over the length scale of the phantom. Although this is a reasonable assumption for a small phantom, the accuracy of the calibration diminishes with phantom size for a given imaging resolution. With a larger phantom that extends beyond the linear region of the gradients, the measured geometry of the phantom, which depends on the gradient profile across the entire phantom, will be overestimated near isocenter and underestimated away from isocenter. This is reflected in the vendor‐adjusted gradient scaling values, which we found to be −0.6%, −1.5%, and +5.8% in x, y, and z relative to the corrected values.

Cyclooctane possesses a number of properties that make it suitable for gradient calibration including isotropic Gaussian diffusion, relatively low diffusivity and high viscosity, and a single proton resonance. These properties were reflected in the highly reproducible diffusion MRI measurements and the excellent fit of the multiple b‐value data. The cyclooctane phantom does not rely on long‐term geometric stability and is simple to build in comparison with geometric phantoms, which typically need tight tolerances requiring specialized 3D printing or fabrication. Errors in diffusion measurements postcalibration were reduced, deviating by <1% with respect to the reference values. Validation with DTI showed that the FA approached zero, as would be expected in an isotropic fluid. Correction factors generated postcalibration were within 0.4% of identity, underscoring the reproducibility of the method.

Independent validation using a grid phantom demonstrated that percentage errors in diffusion measurements were roughly double the errors in geometric measurements, supporting the use of diffusion as a sensitive method for gradient calibration. The errors in postcalibration geometric measurements were also <1% with respect to the high‐resolution reference values. The slotted plate design of the grid phantom meant that reference geometric data could be obtained with an optical flatbed scanner, at a similar resolution as a more expensive micro‐CT scanner. Care was taken to position the grid phantom carefully so that the two plates were aligned with the scanner x‐ and y‐axes. In this study, the grid phantom itself served as additional validation and was not a requirement for the calibration procedure.

The proposed calibration method is also efficient in terms of scan time. Rather than acquire new calibration data for each specific diffusion‐weighted sequence 6, 22, the method presented is non–sequence‐specific and improves accuracy of both diffusion and geometric measurements. In our experience, we found that the gradient scaling was a dominant source of error in DTI. Provided that cross‐terms and imaging gradients are accounted for 16, 19, separate calibration of diffusion gradient strengths across individual diffusion‐weighting directions is unnecessary. We further showed that the correction factors could be calculated in x, y, and z directions in 17 min using NSA = 4 and 5 b‐values for fitting, or 4 min with NSA = 2 and 2 b‐values, with a difference in *α* of < 0.1%.

Central to the calibration is accurate monitoring of temperature and reliance on high‐quality reference diffusivity data. Temperature monitoring systems such as we have adapted, are readily available and are used routinely for physiological monitoring. This system provided real‐time temperature monitoring accurate up to ±0.1°C. In practice, a 60 s sliding window was used to reduce noise in the temperature readings. The 2% higher reference diffusivity, *D*_*r*_(*i*), postcalibration reflects an increase in mean sample temperature of 0.7°C from the precalibration to postcalibration scans, primarily due to heating of the sample during the intervening precalibration DTI scan with high b‐values. However, the calibration scans themselves led to negligible sample heating. Given the continuous temperature measurements, the appropriate *D*_*r*_(*i*) can be calculated for each acquisition based on the sample temperature at any given time. However, when averaging data from multiple repetitions and b‐values, accuracy is improved when temperature fluctuations are minimized. It is thus recommended that all calibration scans be performed successively, without interruption by other scans, particularly scans liable to cause sample heating such as those with short TR, multiple refocusing pulses or strong diffusion gradients. Additional dummy scans may help bring the sample to thermal equilibrium, although this was not found to be a requirement. There are several published reports on the diffusivity of cyclooctane 13, 23, 24, 25. As far as the authors are aware, only the work of Tofts et al 13 reports on the diffusivity over a range of temperatures relevant to the present study.

In addition to errors in gradient scaling, gradient linearity decreases with distance from the isocenter, giving rise to perturbations in the uniformity of k‐space sampling and apparent FOV, and consequently to image distortions. Current methods for correcting gradient nonlinearities include deformation mapping based on high‐resolution geometric information 1, 2, 3, and modeling of the gradient field with spherical harmonics 26, 27, truncated linear distributions 28, and exponentials of power series 25. The use of deformation mapping approaches requires manufacture of typically 3D grid phantoms to high tolerances. Geometric gold standard data are also needed and have typically been acquired from separate CT scanning or from specifications at the time of manufacture. The former requires access to a CT scanner, whereas the latter assumes accurate phantom manufacture and perfect geometric stability of the phantom over time. Where required, identification of landmarks for registration (eg, at grid intersections) may limit the accuracy of corrections to the resolution of the MRI data. Alternatively, modeling approaches have been used in conjunction with either simpler diffusion phantoms or with a priori knowledge of the gradient field. These circumvent the cost of building more complex phantoms but make assumptions about the gradient fields. These assumptions may impose constraints on the situations where such corrections are applicable (eg, where the data are of sufficiently high resolution, and over limited FOVs).

Here, we extended the calculation of the correction factors across the sagittal and coronal planes of the diffusion phantom, enabling calculation of continuous deformations for unwarping the image distortions in x, y, and z. The same deformations improved geometric accuracy in both the diffusion phantom and the grid phantom. Whereas correction for gradient nonlinearity was demonstrated in two orthogonal planes, extending the correction to 3D is straightforward. This would require expansion of Equations (5) and (6) into the slice select direction, and 3D data acquisition at the expense of imaging time. Another consideration is that the relative small diameter of the diffusion phantom here limits the region of support in x and y for calculating correction maps. Ideally, a larger phantom occupying the maximum desired FOV for imaging would be used. We observed that percentage errors in the coordinates of the centroids of the holes in the grid phantom were reduced by up to 20‐fold at regions furthest from the magnet isocenter. Residual errors in these regions may be further minimized with the use of a radiofrequency coil with greater extent of sensitivity in the z‐axis. Critically, the proposed method enables 3D model‐free prospective correction of gradient scaling and retrospective correction of the effects of gradient nonlinearity, without the need for phantoms with high geometric tolerances or access to a separate micro‐CT scanner. The correction is not limited to specific data types and applies over an FOV matching the phantom size.

Whereas image distortions were largely removed following correction for gradient nonlinearity, the ADC remained lower at the ends of the FOV in z. We demonstrated that the accuracy of the ADC measurements could be improved significantly by fitting the diffusion data based on recalculated b‐matrices on a voxel‐wise basis after accounting for the corrected gradient strengths. However, such correction for b‐values will not change the fact that the data in regions of poor gradient linearity will not have been acquired with the same nominal b‐values and may present problems, particularly in quantification of non‐Gaussian diffusion where more precisely defined b‐values are required. The 2D correction data here were acquired in a single slice, with five b‐values, three DW directions, and 100 × 100 μm in‐plane resolution requiring 4 h of scan time. The longer scan time is largely attributed to the use of an SE sequence for high geometric fidelity. The scan time can be readily reduced to under 10 min by acquiring two b‐values and lowering the resolution to 500 × 500 μm in‐plane, taking advantage of the smoothly varying gradient fields, and interpolating the data in postprocessing. In this study, we investigated a range of b‐values up to 2,500 s/mm^2^ as appropriate for models such as DTI and diffusion kurtosis imaging. Based on our previous work in calibrating diffusion spectrum imaging, where we showed that the signal decay of cyclooctane was monoexponential up to 10,000 s/mm^2^ 22, the proposed calibration is expected to be valid across this wider range of b‐values as well.

One consideration is that cyclooctane is flammable. However, the volume of diffusate in the calibration phantom is less than 30 mL. This volume can be reduced further if only correction for gradient scaling is required, as only a few central voxels free of partial volume contamination are required for fitting. For larger FOV coverage, a sturdier wall construction would be required to securely contain the larger volume of cyclooctane.

A limitation of the study is that the linearity of the gradient amplifier response was not investigated explicitly. However, the excellent fit of the multiple b‐value data and the positive results of the geometric validation, where the imaging and diffusion gradient strengths ranged from 3.56 to 628.1 mT/m, suggest that the amplifier linearity was good within this range of gradient strengths. A second limitation is that only one radiofrequency coil was used for calibration. Because different radiofrequency coils may give rise to different eddy current behavior, it could be beneficial to use pulse sequences such as the twice‐refocused SE 29 that minimize eddy currents. In the present study, eddy currents were mitigated by using volume transmit coils with shields built from overlapping slits 30, and were not found to be a major issue.

While the proposed gradient calibration was demonstrated on a preclinical scanner at 9.4T, the method is equally applicable for calibrating gradient systems on clinical scanners at lower field strengths. The key differences of a clinical system compared with the preclinical system employed in this study are the lower gradient strengths, larger FOV, and potential eddy current effects. These require longer diffusion times, more robust phantom construction, and hardware or pulse sequences that minimize eddy currents, respectively, but are otherwise not an impediment to the implementation of the method. Because anatomical and diffusion MRI are ubiquitous in the clinic, improving the accuracy of such measurements over an extended FOV could find widespread clinical application

## CONCLUSIONS

Calibration of the gradient system is important for quantitative MRI. The proposed method provides a simple and efficient means of gradient calibration and correction for gradient nonlinearities in 3D. The method improves accuracy in both geometric and diffusion measurements, without major overhead in phantom fabrication or imaging demands. As the calibration is non–sequence‐specific, it only needs to be performed on a periodic basis as part of a routine MRI quality assurance protocol.
